# Distinct preoperative clinical features predict four histopathological subtypes of high-grade serous carcinoma of the ovary, fallopian tube, and peritoneum

**DOI:** 10.1186/s12885-017-3573-1

**Published:** 2017-08-29

**Authors:** Takuma Ohsuga, Ken Yamaguchi, Aki Kido, Ryusuke Murakami, Kaoru Abiko, Junzo Hamanishi, Eiji Kondoh, Tsukasa Baba, Ikuo Konishi, Noriomi Matsumura

**Affiliations:** 1 Department of Gynecology and Obstetrics, Kyoto University, 54 Kawahara-cho, Shogoin, Sakyo-ku, Kyoto, 606-8507 Japan; 2 Department of Diagnostic Imaging and Nuclear Medicine, Kyoto University, Kyoto, Japan; 3 National Hospital Organization Kyoto Medical Center, Kyoto, Japan

**Keywords:** High-grade serous carcinoma, Ovarian cancer, Subtype, MRI

## Abstract

**Background:**

The Cancer Genome Atlas Research Network reported that high-grade serous carcinoma (HGSC) can be classified based on gene expression profiles into four subtypes, termed “immunoreactive,” “differentiated,” “proliferative,” and “mesenchymal.” We previously established a novel histopathological classification of HGSC, corresponding to the gene expression subtypes: immune reactive (IR), papillo-glandular (PG), solid and proliferative (SP), and mesenchymal transition (MT). The purpose of this study is to identify distinct clinical findings among the four pathological subtypes of HGSC, as well as to predict pathological subtype based on preoperative images.

**Methods:**

We retrospectively assessed 65 HGSC cases (IR: 17, PG: 7, SP: 14, MT: 27) and analyzed preoperative images.

**Results:**

All IR cases originated from either the ovary or fallopian tube (*P* = 0.0269). Significantly more IR cases were diagnosed at earlier stages (*P* = 0.0013), and IR cases displayed lower levels of ascites (*P* = 0.0014), fewer peritoneal lesions (*P* = 0.0080), a sporadic pattern of peritoneal lesions (*P* = 0.0016), a lower incidence of omental cake (*P* = 0.0416), and fewer distant metastases (*P* = 0.0146) compared with the other subtypes. MT cases were more likely to be of peritoneal origin (*P* = 0.0202), presented at advanced stages with higher levels of ascites (*P* = 0.0008, 0.0052, respectively), and more frequently had a diffuse pattern of peritoneal lesions (*P* = 0.0059), omental cake (*P* = 0.0179), and distant metastasis (*P* = 0.0053). A decision tree analysis estimated the histopathological subtypes based on preoperative images, with a sensitivity of 67.3%.

**Conclusions:**

Pathological subtypes of HGSC have distinct clinical behaviors, and preoperative images enable better prediction of pathological subtype. These findings may lead to individualized treatment plans if the effect of treatment based on the HGSC subtype is elucidated.

**Electronic supplementary material:**

The online version of this article (10.1186/s12885-017-3573-1) contains supplementary material, which is available to authorized users.

## Background

Ovarian carcinoma is the most common cause of gynecologic cancer death and the fifth leading cause of cancer deaths in women in the United States [1]. High-grade serous carcinoma (HGSC), accounting for 68% of ovarian carcinoma cases, is usually diagnosed at an advanced stage and has a poor prognosis [2]. Chemotherapy with a taxane- and platinum-based regimen is typically provided after debulking surgery, and 75% of high-grade serous ovarian carcinoma cases respond to initial treatment [3]. However, many patients experience recurrence and ultimately succumb to the disease.

Serous carcinoma is the most common histological subtype of primary peritoneal cancer and fallopian tube cancer, as well as epithelial ovarian cancer [4]. Traditionally, serous epithelial tumors in the ovaries, primary fallopian tubes, and peritoneum have all been approached as primary epithelial ovarian tumors in clinical and research settings because of their shared clinical behavior and treatment [5]. However, in practice, HGSC seems to be a heterogeneous disease, because cases have a diversity of clinical features, therapeutic responses, and prognoses. Analysis of gene expression microarray data from The Cancer Genome Atlas (TCGA) project revealed that HGSC could be classified as one of four gene expression subtypes: “immunoreactive,” “differentiated,” “proliferative,” or “mesenchymal” [6, 7]. These sub-classifications display distinct prognoses and sensitivities to chemotherapy [7–9]. We recently established four histopathological classifications of HGSC that correlate with the TCGA gene expression subtypes and prognoses: immune reactive (IR), which is defined by lymphocytes surrounding and infiltrating the malignant tissue; papillo-glandular (PG), which is defined by a papillary architecture; solid and proliferative (SP), which is defined by a solid growth pattern; and mesenchymal transition (MT), which is defined by a remarkable desmoplastic reaction [10]. Of these histopathological sub-classifications, the MT subtype has the worst prognosis, and the IR subtype has the most favorable prognosis. Therefore, the exact diagnosis of MT and IR subtypes is important for the clinical management of HGSC. However, diagnosis of these subtypes requires biopsy of the tumor, which is located in the abdominal cavity and can be difficult to access.

Although the mesenchymal gene expression subtype of ovarian tumors is accompanied by mesenteric infiltration and diffuse peritoneal disease on computed tomography (CT) imaging, it was previously not possible to define conclusive association of the four pathological subtypes with distinct clinical features [11]. The first purpose of this study is the identification of distinct clinical features among the four pathological subtypes of HGSC. The second aim is to predict pathological subtype using preoperative factors, including images. Prediction of pathological subtypes of HGSC will enable clinicians to estimate chemosensitivity and prognosis, allowing the administration of individualized therapies without performing exploratory laparotomy or laparoscopy.

## Methods

### Eligibility criteria

This retrospective study was approved by the institutional ethics committee. Patients were included if they: (a) underwent primary debulking surgery or exploratory laparoscopy and were newly diagnosed histologically with HGSC of the ovary, fallopian tube, or peritoneum between 2005 and 2014 at the Kyoto University Hospital, and (b) underwent magnetic resonance (MR) imaging of the pelvis and CT of the neck, chest, abdomen and pelvis prior to initial treatment. Sixty-five patients, all of whom provided informed consent, satisfied the eligibility criteria and were included in this study.

### Patient characteristics and pathological review

**Fig. 1:**
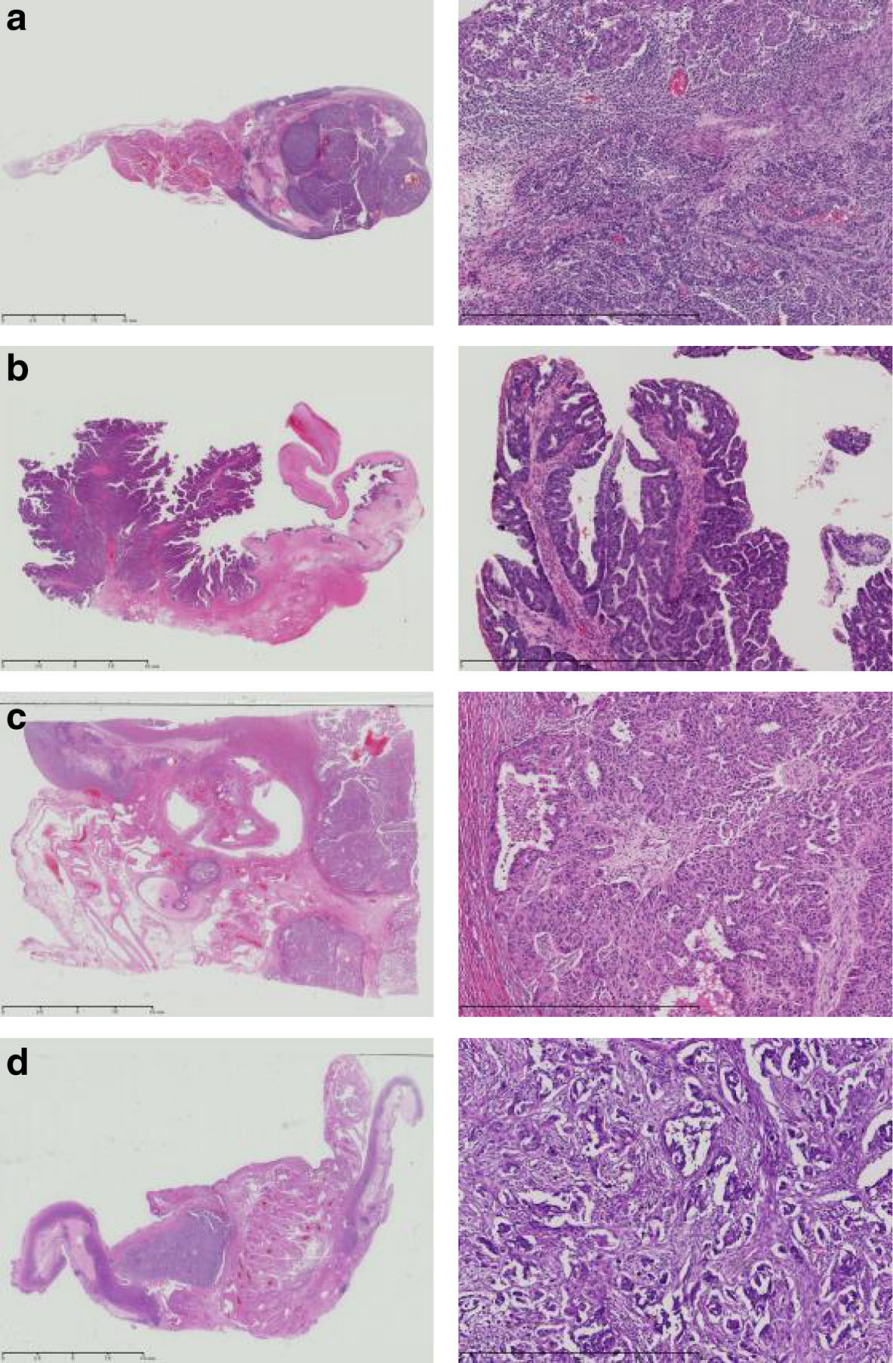
Four pathological subtypes of HGSC. **a**) Immune reactive (IR): infiltration by numerous lymphocytes with a smooth invasive front. **b**) Papillo-Glandular (PG): papillary architecture. **c**) Solid and Proliferative (SP): a solid growth pattern. **d**) Mesenchymal Transition (MT): a remarkable desmoplastic reaction and a scattered invasion or labyrinthine pattern. Left figures are loupe images, and right images are at 200× magnification

We classified HGSC into four subtypes using our previously described algorithm (Fig. 1) [10]. At least four blinded pathologists and gynecologists determined the pathological subtypes of HGSC cases. We used the International Federation of Gynecology and Obstetrics classification for ovary, fallopian tube, and primary peritoneal carcinoma [12].

### Image analysis

Pretreatment imaging was obtained within one month before starting initial treatment. Firstly, a gynecologist and a radiologist specializing in gynecological diagnostic imaging who were blinded to the patients’ histopathological subtypes independently evaluated all MR and CT images. When their image interpretations differed, the final decision was settled by discussion. Secondly, another radiologist also specializing in gynecological diagnostic imaging evaluated all of the images. In cases of differing interpretations, consensus was reached by discussion.

**Fig. 2:**
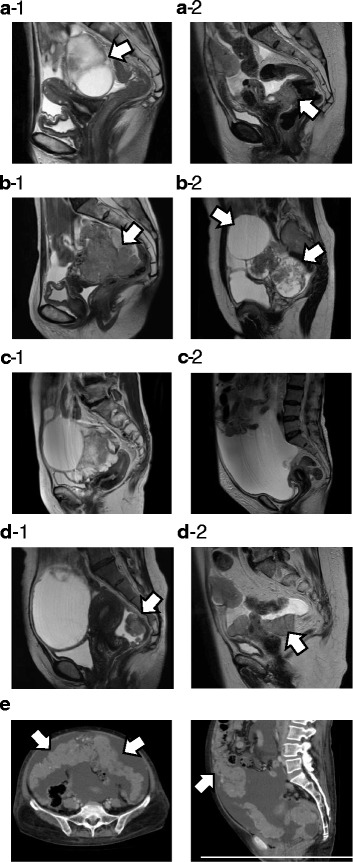
Representative magnetic resonance (MR) imaging and computed tomography (CT) findings. **a**) Location of the main tumor by MR imaging. a-1) Ovary or fallopian tube (arrow). a-2) Peritoneum (arrow). **b**) Morphology of the main tumor by MR imaging. b-1) Solid: more than 50% of the main tumor is solid (arrow). b-2) Cystic: more than 50% of the main tumor is cystic (arrow). **c**) The amount of ascites by MR imaging. c-1) Small amount: ascites within the pelvis. c-2) Large amount: ascites beyond the pelvis. **d**) Pattern of peritoneal dissemination by MR imaging. d-1) Sporadic pattern: single or multiple nodules scattered sporadically in the peritoneum (arrow). d-2) Diffuse pattern: numerous nodules or masses spread diffusely along the peritoneum (arrow). **e**) Omental cake by CT: abnormally thickened greater omentum (arrow)

The following features were evaluated by CT and MR imaging:

1. the location of the main tumor as ovary/fallopian tube or peritoneum (peritoneum included any cases without an ovarian or fallopian tube mass) (Fig. 2a)
2. the morphology of the main tumor as solid (more than 50% of the main tumor was solid) or cystic (more than 50% of the main tumor was cystic) (Fig. 2b)
3. the amount of ascites as small (within the pelvis) or large (beyond the pelvis) on MR imaging (Fig. 2c)
4. the presence/absence of peritoneal dissemination
5. the morphological pattern of peritoneal dissemination as sporadic (single or multiple nodules scattered sporadically in the peritoneum) or diffuse (numerous nodules or masses spread diffusely along the peritoneum) on CT or MR imaging, according to the criteria defined previously [11] (Fig. 2d)
6. the presence/absence of omental cake, defined as an abnormally thickened omentum (Fig. 2e)
7. the presence/absence of lymph node metastasis on CT (lymph nodes more than 10 mm in the short axis were considered metastatic)
8. the presence/absence of distant metastasis on CT

The decision tree was generated using Weka, a publically available data mining software program (http://www.cs.waikato.ac.nz/ml/weka/).

### Statistical methods

All statistical analyses were performed with EZR (Saitama Medical Center, Jichi Medical University, Saitama, Japan), which is a graphical user interface for R (The R Foundation for Statistical Computing, Vienna, Austria). More precisely, it is a modified version of the R commander designed to add statistical functions frequently used in biostatistics [13]. Fisher’s exact probability test was used to examine the relationships between the clinical parameters stated above and the histopathological subtypes of HGSC. *P*-values <0.05 were considered significant.

## Results

### Distinct clinical findings among the four pathological subtypes of HGSC

The 65 cases included 17 IR, 7 PG, 14 SP, and 27 MT histopathological subtypes. Table 1 shows the association between clinical findings and the four histopathological subtypes. Further details of these findings are shown in Additional file 1: Table S1. More IR cases were diagnosed at earlier stages compared with the other subtypes (8/17 vs. 4/48, *P* = 0.0013), and more MT cases were diagnosed at advanced stages (27/27 vs. 26/38, *P* = 0.0008). As for the location of the main tumor on MR imaging (Fig. 2a), all of the IR cases had a main tumor in the ovaries or fallopian tubes, and this was less common in the other subtypes (17/17 vs. 36/48, *P* = 0.0269), while significantly more MT cases had a main peritoneal lesion compared with the other subtypes (9/27 vs. 3/38, *P* = 0.0202). Although the morphology of the main tumor was not significantly associated with histopathological subtype, PG cases tended to exhibit cystic tumors (Fig. 2b). The amount of ascites on MR imaging (Fig. 2c) was significantly lower in IR cases compared with the other subtypes (14/17 vs. 17 /48, *P* = 0.0014) and higher in MT cases (20/27 vs. 14/38, *P* = 0.0052). The frequency of peritoneal lesions on MR imaging was significantly lower in IR cases compared with the other subtypes (8/17 vs. 40/48, *P* = 0.0080) and higher in MT cases (26/27 vs. 22/38, *P* = 0.0004). Peritoneal lesions on MR imaging (Fig. 2d) were more frequently sporadic in IR cases than in other subtypes (6/8 vs. 6/40, *P* = 0.0016) and more frequently diffuse in MT cases than in other subtypes (24/26 vs. 12/22, *P* = 0.0059). Omental cakes were observed on CT (Fig. 2e) significantly less frequently in IR cases compared with other subtypes (7/17 vs. 34/48, *P* = 0.0416) and more frequently in MT cases (22/27 vs. 19/38, *P* = 0.0179). Lymphadenopathy on CT was detected significantly more frequently in SP cases compared with the other subtypes (9/14 vs. 14/51, *P* = 0.0243), while no radiographic lymphadenopathy was detected in PG cases (0/7 vs. 23/58, *P* = 0.0452). IR cases showed no distant metastases on CT (0/17 vs. 13/48, *P* = 0.0146). Additionally, none of the 7 PG cases showed distant metastases on CT. On the other hand, MT cases showed significantly more distant metastases on CT (10/27 vs. 3/38, *P* = 0.0053). These findings are summarized in Table 2.

**Table 1:** Clinical features among the four pathological subtypes of HGSC

Pathological subtype		IR	PG	SP	MT
FIGO stage					
Early (Stage I & II)		8	2	2	0
Advanced (Stage III & IV)		9	5	12	27
*P* value		0.0013*	0.6043	1.0000	0.0008*
Location of main tumor in MRI					
Ovary or Fallopian tube		17	5	13	18
Peritoneum		0	2	1	9
*P* value		0.0269*	0.6043	0.4364	0.0202*
Morphology of main tumor in MRI					
Solid		11	4	11	21
Cystic		6	3	3	6
*P* value		0.5299	0.3853	0.7402	0.5748
The amount of ascites in MRI					
Small		14	5	5	7
Large		3	2	9	20
*P* value		0.0014*	0.2440	0.3750	0.0052*
Peritoneal lesion in MRI					
Presence	Negative	9	4	3	1
	Positive	8	3	11	26
*P* value		0.0080*	0.0700	0.7448	0.0004*
Pattern	Sporadic	6	0	4	2
	Diffuse	2	3	7	24
*P* value		0.0016*	0.5629	0.4304	0.0059*
Omental cake in CT					
Negative		10	4	5	5
Positive		7	3	9	22
*P* value		0.0416*	0.4080	1.0000	0.0179*
radiographically enlarged lymph nodes in CT					
Negative		9	7	5	21
Positive		8	0	9	6
*P* value		0.2554	0.0452*	0.0243*	0.0717
Distant metastasis in CT					
Negative		17	7	11	17
Positive		0	0	3	10
*P* value		0.0146*	0.3287	1.0000	0.0053*

*P* value: Fisher’s exact test, comparing between each subtype and the other subtypes, * *p* < 0.05

**Table 2:** Summary of clinical features among the four histopathological subtypes of HGSC

Pathological subtype	IR	PG	SP	MT
FIGO stage	Early	Advanced	Advanced	Advanced
Location of main tumor	Ovary or Fallopian tube	Ovary or Fallopian tube	Ovary or Fallopian tube	Peritoneum
Ascites	↓↓		↑	↑↑
Peritoneal lesion	↓↓		↑	↑↑
	sporadic	diffuse	diffuse	diffuse
Omental cake	↓↓		↑	↑↑
LN swelling	↑	↓↓	↑↑	
Distant metastasis	↓↓	↓	↓	↑↑
Prognosis	favorable			poor

Abbreviation: ↑↑: significantly more ↑: more ↓: less ↓↓: significantly less

### Prediction of pathological subtypes using pre-treatment clinical findings

**Fig. 3:**
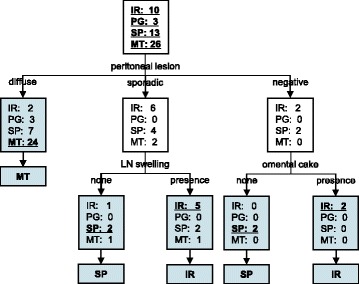
Algorithm for pretreatment prediction of the four histopathological subtypes. Algorithm for pretreatment prediction of the four histopathological subtypes using decision tree analysis. MT: mesenchymal transition, IR: immune reactive, SP: solid and proliferative, PG: papillo-glandular

We conducted a decision tree analysis for HGSC cases suggestive of stage III and IV to predict pathological subtype preoperatively using suggestive origin (location of main tumor), morphology of the primary tumor, amount of ascites, presence of omental cake, presence and pattern of peritoneal disease, radiographically enlarged lymph nodes, and presence of distant metastasis (Fig. 3). Thirteen cases that were suggestive of stage I and II because of the small amount of ascites and absence of omental cake, peritoneal dissemination, radiographically enlarged lymph nodes, and distant metastasis were excluded from the decision tree analysis. A decision tree with the 52 remaining samples (IR: 10, PG: 3, SP: 13, and MT: 26 cases) that were suggestive of stage III and IV indicated that the pattern of peritoneal dissemination, radiographically enlarged lymph nodes, and presence of omental cake were useful identifiers for sub-classification of HGSC. Of 52 patients, 24 out of 36 cases with diffuse pattern of peritoneal lesion were the MT subtype. Of the other 16 cases, a sporadic pattern of peritoneal dissemination identified 2 MT, 6 IR, and 4 SP cases. Of the 12 cases with a sporadic pattern of dissemination, 4 did not exhibit radiographically enlarged lymph nodes, two of which were the SP subtype. Of the 8 cases with a sporadic pattern of peritoneal dissemination and radiographically enlarged lymph nodes, 5 cases belonged to the IR subtype. Of the 4 cases without peritoneal lesions, 2 cases without omental cake were the SP subtype and the other two cases with omental cake showed the IR subtype. This algorithm in total had a sensitivity of 67.3%. The sensitivities of diagnosis were 70.0% (7/10), 0% (0/3), 30.8% (4/13) and 92.3% (24/26), for the IR, PG, SP, and MT subtypes, respectively.

## Discussion

This investigation clearly shows that the pathological subtypes of HGSC exhibit distinct clinical behaviors (Table 2). Our previous study revealed that these pathological subtypes are statistically correlated with the previously defined TCGA gene expression subtypes, and that the MT subtype is a poor prognostic factor, while the IR subtype is a favorable prognostic factor [10]. Although, in general, high-grade serous ovarian, fallopian tube, and peritoneal cancer are considered a single clinical entity because of their shared clinical behavior and treatment, some studies have found a significantly poorer survival or a non-significant trend of poorer survival for primary peritoneal tumors compared with ovarian tumors [5]. Vargas et al. suggested that mesenteric infiltration and diffuse peritoneal disease on CT are associated with the mesenchymal gene expression subtype and shorter progression-free survival [11]. These reports are compatible with our findings that the MT subtype, which had the poorest outcome, included significantly more diseases of peritoneal origin and omental cake.

Feigenberg et al. suggested that advanced stage HGSC cases presenting with low-volume ascites are associated with the upregulation of immune-related genes, more immune cells infiltrating the tumor, and better clinical outcomes [14]. In addition, Baek et al. reported that patients with stage III disease solely by lymph node metastasis showed even better outcomes than did those with stage III disease with peritoneal dissemination [15]. Our study indicated that the IR subtype tends to show lower volume ascites, less peritoneal dissemination, and more radiographic lymphadenopathy on CT than other subtypes. Our decision tree analysis also showed that IR cases with advanced stage exhibit radiographically enlarged lymph nodes and a small amount of ascites on CT. The IR subtype has a favorable prognosis [6, 7, 9, 16], which may be owing to these advanced cases having a lower volume of ascites, less peritoneal dissemination, and radiographically smaller lymph nodes. Vargas et al. reported that ovarian mass morphology in high-grade serous ovarian cancer on CT imaging was not definitely associated with gene expression subtype [11]. In our study, morphology of the main tumor in MR imaging was not specifically associated with any of the four pathological subtypes.

Additionally, this study may be useful in determining the initial chemotherapeutic regimen for individualized treatment. Studies indicate that the four pathological subtypes show different patterns of anticancer drug sensitivity. Recently, Symeonides et al. suggested that distinct molecular subgroups of high-grade serous ovarian cancer respond very differently to bevacizumab [8]. In this analysis, the two proangiogenic subgroups, which represent non-IR subtypes in this study, had worse overall survival but included all the patients who benefited from bevacizumab. The immune subgroup had a superior prognosis, but bevacizumab had a detrimental effect in these patients. Therefore, it is likely that the pathological subtypes may be biomarkers for bevacizumab benefit and resistance. Our previous study suggested that the mesenchymal subtype may be particularly sensitive to taxanes and resistant to carboplatin [17]. Because dose-dense paclitaxel and carboplatin (TC) therapy, which gives more weight to paclitaxel than conventional TC chemotherapy, confers a more favorable prognosis compared to conventional TC for HGSC, chemotherapy with dose-dense TC may be beneficial for patients with the MT subtype. With regard to the molecular features, George et al. suggested that breast cancer susceptibility gene 1 (*BRCA1*) disruptions are associated with the immunoreactive molecular subtype of HGSC [18]. Furthermore, Soslow et al. implied that there is a positive association between tumor-infiltrating lymphocytes and BRCA1 loss in HGSC [19]. These findings indicate that the IR subtype has a likelihood of benefiting from poly(ADP-ribose) polymerase inhibitor use [20].

Recently, neo-adjuvant chemotherapy (NAC) has been shown to be a valuable alternative treatment for patients with advanced epithelial ovarian cancer who are not amenable to primary optimum surgery. NAC provides a higher rate of optimal cytoreduction and equivalent survival with less invasive surgery and reduced morbidity compared to conventional therapy [21, 22]. Exploratory laparotomy or laparoscopy is necessary to select the anti-cancer agents for NAC based on the pathological and TCGA gene expression subtypes. However, exploratory surgery is contraindicated in some advanced cases, such as in the presence of a large amount of ascites or pleural effusion. Preoperative estimation of the pathological subtype using imaging possibly allows the patients for whom the surgery is contraindicated to start NAC immediately using the optimal regimen.

There are several limitations to this study. The limited number of cases may have caused statistically insignificant results, particularly in the PG subtype. A decision tree using pattern of peritoneal dissemination, the existence of radiographic lymphadenopathy, and omental cake did not predict the PG subtype, whereas the IR and MT subtypes were identified with 70.0% and 92.3% sensitivity, respectively. Additionally, our findings were not validated using external datasets. However, it is meaningful that this decision tree can diagnose the IR and MT subtypes, which show the most favorable and poorest prognosis, respectively. Further studies should be performed with a larger sample size, and the accuracy of this algorithm on the effect of treatment needs to be validated in a prospective manner.

## Conclusions

We revealed four histopathological subtypes of HGSC of the ovary, fallopian tube, and peritoneum, demonstrating distinct clinical features and pretreatment images that enable estimation of the histopathological subtypes. These findings have the potential to help in determining an initial treatment strategy for individualized treatment.

## Additional file

Additional file 1: Table S1. Clinical findings of individual cases. Individual findings include pathological subtype, stage, suggestive origin (location of primary tumor), morphology of the primary tumor, amount of ascites, presence of omental cake, presence and pattern of peritoneal disease, radiographically enlarged lymph nodes, and presence of distant metastasis. (XLSX 13 kb)

## Abbreviations

BRCA1: breast cancer susceptibility gene 1
CT: computed tomography
HGSC: high-grade serous carcinoma
IR: immune reactive
MR: magnetic resonance
MT: mesenchymal transition
NAC: neo-adjuvant chemotherapy
PG: papillo-glandular
SP: solid and proliferative
TC: paclitaxel and carboplatin
TCGA: The Cancer Genome Atlas
